# Effect of dipole polarization orientation on surface plasmon coupling with green emitting quantum wells by cathodoluminescence

**DOI:** 10.1039/c8ra01859f

**Published:** 2018-05-03

**Authors:** Yulong Feng, Zhizhong Chen, Chengcheng Li, Yifan Chen, Jinglin Zhan, Yiyong Chen, Jingxin Nie, Fei Jiao, Xiangning Kang, Shunfeng Li, Qi Wang, Tongjun Yu, Guoyi Zhang, Bo Shen

**Affiliations:** State Key Laboratory for Artificial Microstructure and Mesoscopic Physics, School of Physics, Peking University Beijing 100871 China zzchen@pku.edu.cn +86 13167388462; A State Key Laboratory of Nuclear Physics and Technology, School of Physics, Peking University Beijing 100871 China; Dongguan Institute of Optoelectronics, Peking University Dongguan 523808 Guangdong China

## Abstract

Ag nanoparticles (NPs) are fabricated on the cross-section of green emitting quantum wells (QWs). The effect of the dipole polarization orientation on the localized surface plasmon (LSP)-QW coupling can be studied by setting the incident direction of the electron beam parallel to the plane of the QWs. Cathodoluminescence (CL) measurements on the QWs show that the intensity with the Ag NPs is enhanced 6.1 times compared with that without the Ag NPs. Total energy loss profiles for an electron beam in the GaN and Ag NP are accurately simulated using a Monte Carlo program (CASINO). The orientations of the in-plane dipoles in the QWs can vary from 0° to 360°. Through a two-step simulation process using the three-dimensional (3D) finite difference time domain (FDTD) method, the weighted average of CL intensities are simulated for QWs with the Ag NPs. The simulation results agree well with the experimental results. Lastly, the dipole orientation dependent LSP-QW coupling process is discussed.

## Abstract 2

Ag nanoparticles (NPs) are fabricated on the cross-section of green emitting quantum wells (QWs).

## Graphical abstract

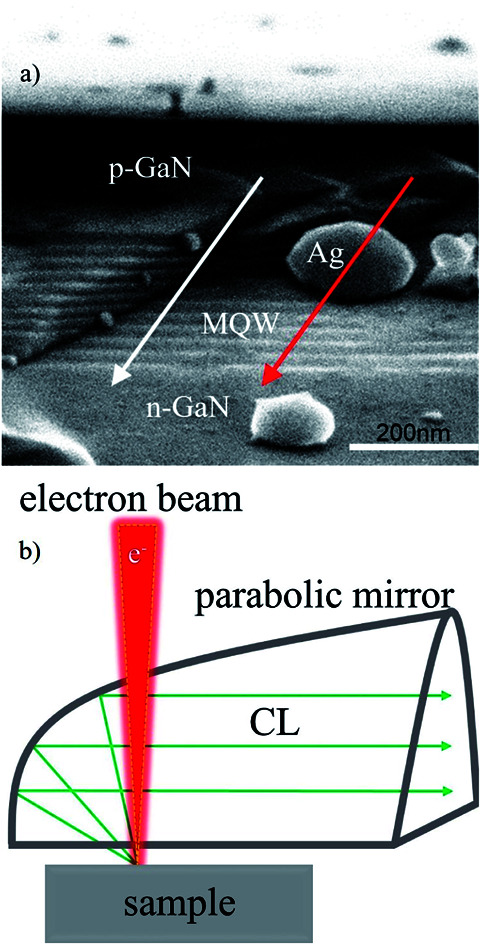

## Introduction

The well-known “green gap” has been a big issue for several years.^1^ Surface plasmon (SP) is one of the effective techniques to avoid the key problems of poor crystalline quality and high polarization fields in the gallium nitride (GaN)-based green light emitting diodes (LEDs).^2–8^ According to Fermi's golden rule, the radiative recombination rate in quantum wells (QWs) is proportional to the density of state (DOS) and local electric field of SP modes.^9^ The carriers in the QWs near the metal's surface will be quickly radiatively recombined through the high local electric field^2,4^ or a new recombination channel provided by SP with high DOS.^6–8^ The nonradiative recombination, due to the defects in the InGaN QWs, is relatively little affected because of the large enhancement of radiative recombination. Moreover, the quantum-confined Stark effect (QCSE) caused by the polarization field is weakened through SP-coupled green LEDs.^5^ Far-field SP coupling is also used to enhance the electroluminescence (EL) for organic green LEDs.^10^

However, due to the high ohmic loss inside the metals^11^ or nonradiative high-order SP modes,^12^ light emission enhancement is very hard to achieve for SP-coupled LEDs when the initial internal quantum efficiency (IQE) is high.^8^ The SP modes are generated by excitons instead of photons or phonons, whereas the extraction of light from the SP modes require the establishment of suitable wave-vector matching conditions at the interface using nanostructures. Recently, Ag nanoparticles (NPs) and the very thin p-GaN spacers between the metallic NPs and QW layer were adopted, and about 2 times EL intensity enhancements were achieved.^6,13^ The doped graphene induced SPs are also used in the EL enhanced LEDs.^14^ It is also found the light emission enhancement is determined by the size, shape, arrangement of the metallic NPs.^4,8,15,16^ Both the spontaneous emission rate and IQE are enhanced greatly by the SP gap modes, where the radiators are placed between an Ag nanocube and Au film with random polarization orientations from 60° to 90°.^17^ Zhu *et al.* etched nanorod LED through 1–2 QWs and put the Ag NPs into the spacers between the nanorods.^7^ The radiating dipoles in the first 1–2 QWs may have different orientations with respect to the Ag NPs. However, the polarization orientation between the radiator and metallic NPs seems not to be paid much attention to.

Since the radiating dipoles in an InGaN/GaN QW mainly lie in the QW plane based on theoretical studies, only the in-plane dipoles are considered in most studies when the metallic NPs located on or beneath the QWs where dipoles are mainly parallel to the bottom surface of the metallic NPs.^5,18^ In numerical simulations, the radiating dipoles non-parallel to the QW plane are also studied.^2,19,20^ Yang *et al.* classified the radiating dipoles as radial and orbital ones according to the orientation with respect to the Ag nanosphere.^19,20^ The radial and orbital dipoles couple with the localized SP (LSP) dipole and higher-order resonance modes, respectively. They found both the LSP dipole and higher order resonance modes could make significant contributions to the radiated power enhancement of the LSP-QW coupling system.^19^ The resonant wavelength is longer and the phase retardation effect is more significant for dipole modes coupling.^2^ It is quite different from the pure dissipation modes of higher order SPs calculated by quasi-static approximation approach.^12^ Although the radial dipole show stronger coupling to SP dipole mode, the radial dipole structures is difficult to fabricate.^7,17^ The simulation results need more experimental evidences.

On the other hand, according to the recent reports, cathodoluminescence (CL) has been performed on the Ag NPs in a scanning electron microscope (SEM) setup.^21–23^ With the combination of the ultrahigh spatial resolution of the electron beam (e-beam) and broadband optical sensitivity, optical process and nanometer-sized features of the Ag NPs can be resolved. The octupolar LSP mode can be excited *via* high-energy e-beam.^22^ There are also some reports on the Ag NPs-QW structure excited by e-beam.^24,25^ However, they did not concern the SP modes induced by e-beam and its effect on the QWs under the metallic NPs. Few results on the different polarization orientation radiators coupling with SPs are demonstrated by CL measurements.

In this work, Ag nanoparticles (NPs) were fabricated on the cross-section of the green emitting quantum wells (QWs). The effect of the dipole polarization orientation on the LSP-QW coupling can be studied by setting the incident direction of the electron beam parallel to the plane of the QW. CL line scanning measurements with and without the Ag NPs were performed. Monte Carlo program (CASINO) was used to model the total energy loss profiles for the electron interactions in the GaN and Ag NP.^26^ To illustrate the dipole orientation dependent LSP-QW coupling mechanism, a 3D FDTD (Lumerical FDTD Solutions v8.17, Vancouver, BC, Canada)^27^ simulation procedure corresponding to the CL measurement was also carried out.

## Experimental

**Fig. 1:**
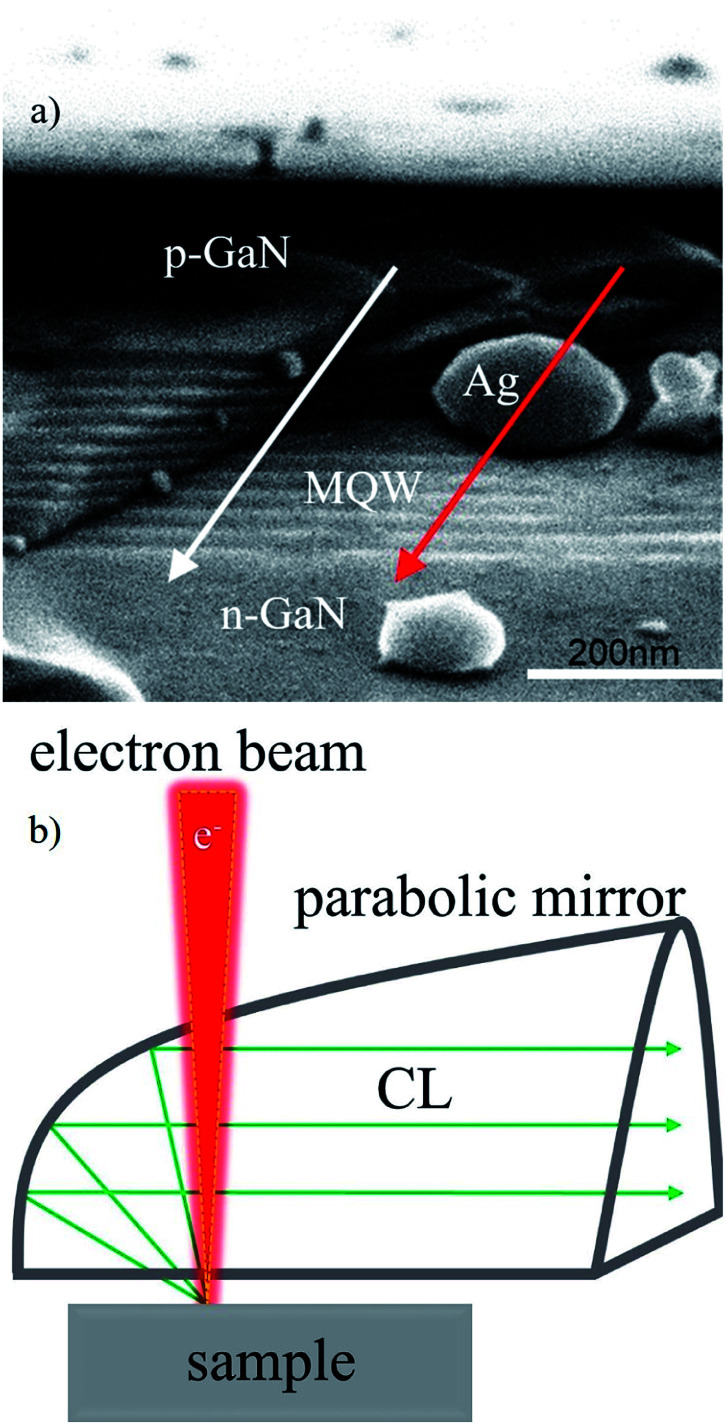
(a) Cross-sectional SEM image of the LSP-QW coupled sample. The Ag NP overlaps the region of QWs/p-GaN. The major and minor axes of the Ag NP are 200 and 120 nm, respectively. (b) Schematic setup for the CL measurement.

The epitaxial structure of GaN-based green LED at 545 nm used in the experiment was grown by metal organic chemical vapor deposition (MOCVD) on a *c*-plane sapphire substrate. The LED structure consisted of 180 nm-thick p-GaN, 10 pairs of InGaN/GaN (2.5 nm/17.5 nm) QWs and a 4 μm n-GaN layer. The Ag NPs embedded in the hexagonal photonic crystals (PhCs) array holes were fabricated in a green LED by nanoimprint and lift-off techniques, as described in ref. 15 in detail. The cross sectional specimens were prepared by cleaving the sample for scanning electron microscope (SEM) and CL measurement. Some Ag NPs were found scattered from the PhC holes and located in the QWs region in the cross-section. As shown in Fig. 1(a), the Ag NP was ellipsoidal, whose major and minor axes are 200 and 120 nm, respectively. In this geometry, the orientation of the dipoles in the QW-planes can be changed from 0° to 180°, including the radial and orbital components with respect to Ag NPs.

A Gatan MonoCL4 system (Gatan, Pleasanton, CA, USA) was equipped in a SEM platform for CL measurement, as shown in Fig. 1(b) schematically. The e-beam was highly focused and directed onto the surface of the sample. The injected electrons excited the cathodoluminescence. The emitted light was collected by a parabolic mirror and separated into its component wavelength by a monochromator and finally detected by a charge-coupled device (CCD). The sample was placed at the focal plane of the parabolic mirror to make sure that most of the emitted light can be collected. The spectral resolution was less than 0.5 nm. Typically, by scanning the e-beam along a direction and measuring the intensity and wavelength point by point, a high-resolution line scanning of the optical activity of the specimen can be obtained. As shown in Fig. 1(a), the e-beam (electron energy of 10 keV) was moved along the white and red lines to measure the CL spectrum at each point. The scanning step size was set to be 16 nm. The red line scanned through the Ag NP from p-GaN to the QWs (labelled Ag), while the white parallel line scanned through a region without the Ag NPs (labelled woAg). When the e-beam impinged on the Ag NP, the collective electron oscillations in a metallic particle, namely the LSP modes, were induced and responsible for the light emission excited in the QWs underneath.

## Results and discussion

**Fig. 2:**
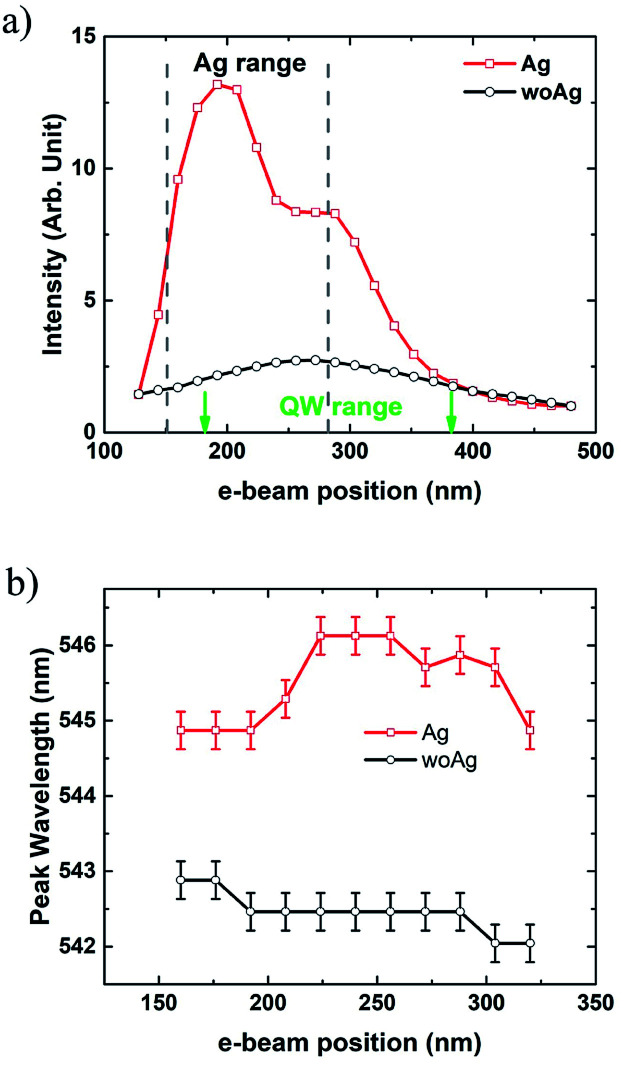
(a) The peak intensities and (b) wavelengths in CL line scanning. The red and black curves correspond to the red and white lines in Fig. 1(a). The horizontal coordinates indicate the distance from the surface of p-GaN. The legends of “Ag” and “woAg” corresponds to the red and white lines in Fig. 1(a). The spectral resolution of Gatan MonoCL4 system is less than 0.5 nm.

Fig. 2 shows the peak intensities and wavelengths in the CL line scanning along the directions marked in Fig. 1(a). The red and black curves correspond to the red and white lines in Fig. 1(a). The horizontal coordinates indicate the distance from the surface of p-GaN. The grey dashed lines in Fig. 2(a) denote the range of the Ag NP from 150 to 270 nm away from the surface of p-GaN. The green arrows indicate the range of the QWs from 180 to 380 nm. When the electron impinging points are not in the range of the Ag NP or QWs, CL peak intensities are similar for both lines. When the e-beam approaches to the Ag NP, the intensity rises rapidly. The maximum CL peak intensity is obtained near the top of the Ag NP, which is enhanced 6.1 times compared with that without the Ag NP at the same position. The CL intensity enhancement is attributed to the LSP induced by high energy electron beam and/or excited QWs. Then the intensity drops and remains relatively unchanged from 240 to 300 nm. When the e-beam leaves the Ag NP, the intensity decreases exponentially from 270 to 370 nm. After 370 nm away from the p-GaN surface, the two lines are almost overlapped. When the e-beam scans across the Ag NP, CL enhancement and the exponential decay can be attributed to LSP-QW coupling.^11^ Besides, CL peak wavelengths near the Ag NP along the two lines are shown in Fig. 2(b) with a spectral resolution less than 0.5 nm. An obvious redshift of about 3 nm can be observed in the Ag NP region compared with that without the Ag NP at the same position. Redshift becomes even larger just as the e-beam scans from the top to the side of the Ag NP. The redshift reduces when the e-beam leaves away from the Ag NP. As to the black curve for the scanning without the Ag NP, no redshift can be observed. The blueshift of 1 nm may be caused by the indium content or strain fluctuations in different QWs. When the LSP resonance spectrum overlaps the emission spectrum, the intensity of the overlapping area will be enhanced.^8^ Due to the large size of the Ag NP, the resonance wavelength may be long.^28^ Therefore, the redshift could be observed when the LSP-QW coupling occurs.

Although the line scanning CL results can be well explained qualitatively by LSP-QW coupling, there are still many problems to be resolved. The height of the Ag NPs is too large to be penetrated for an electron with 10 keV energy. Thus, LSP excited by the e-beam needs to be further studied. Since the bottom surface of the Ag NP is perpendicular to the QW-plane, the polarization orientation of a dipole radiator (QW) can be changed arbitrarily with respect to the Ag NP. The effect of the dipole orientation on the light emission is not clear yet. When changing the e-beam impinging position on the sample, the intersecting volume of QWs is also modified.

**Fig. 3:**
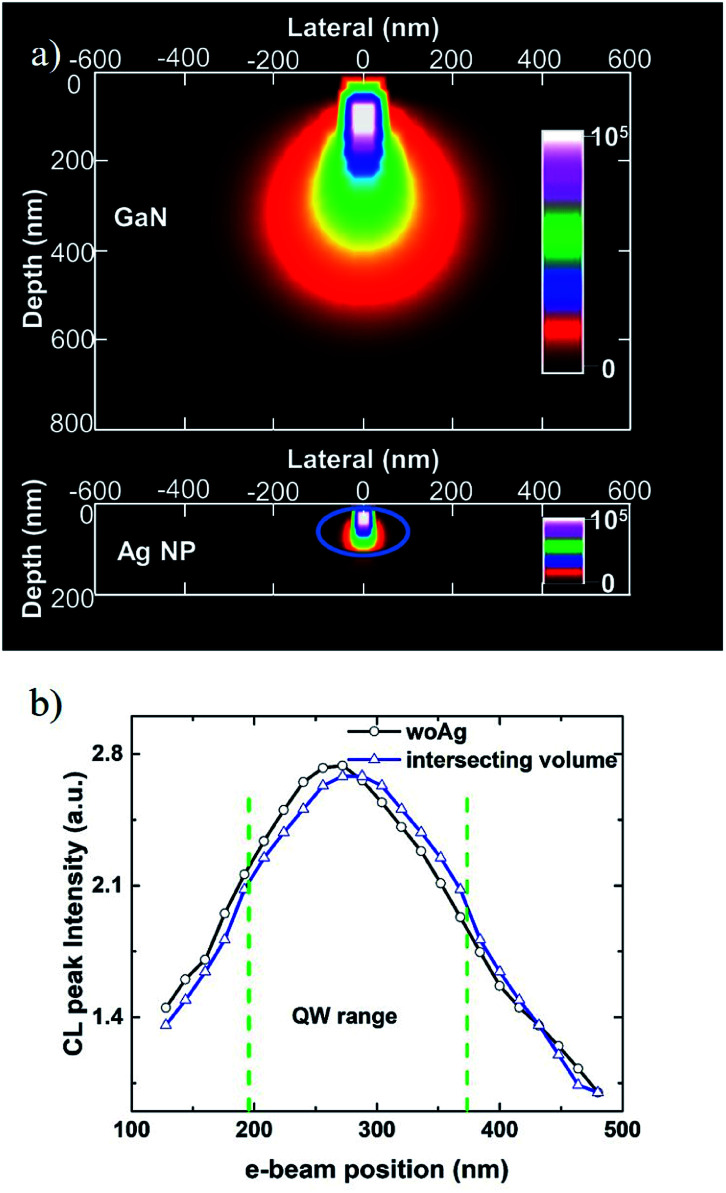
(a) Total energy loss profile simulated (lateral and depth) by CASINO for the GaN and Ag NP respectively. (b) The peak intensity of the CL line scanning through the QWs (black) and the result fitted by intersecting volume assumption (blue).

Here the total energy loss profiles (depth and lateral) generated by an e-beam was calculated by CASINO in the GaN and Ag NP.^26^ To keep consistent with CL measurement, the electron energy is set to be 10 keV. The number of simulated electrons is 10^6^ which is large enough to maintain the accuracy and validity. As shown in Fig. 3(a), more than 80% of the electron energy in the GaN is restricted within a radius (lateral) of about 150 nm while the electrons can hardly penetrate through the Ag NP (the blue ellipse). Hence, it is reasonable to assume that the interactive volume of electrons in the GaN is a spheroid with a radius of 150 nm. As the e-beam moves, the CL intensity changes because of the interactive volume changing. Since CL intensity is proportional to the interactive volume that intersects with QW layers, CL line scanning without Ag NP along white line in Fig. 1(a) can be calculated. As shown by the blue curve in Fig. 3(b), the intensity fitted by the intersecting volume agrees well with the experimental result, indicating the validity of approximating CL generation through the electron interaction volume by the total electron energy loss profile. As to the Ag NP where the electrons can hardly penetrate through, the CL intensity enhancement at the top of the Ag NP should be attributed to the LSP induced by e-beam^22^ coupling to the QWs.

**Fig. 4:**
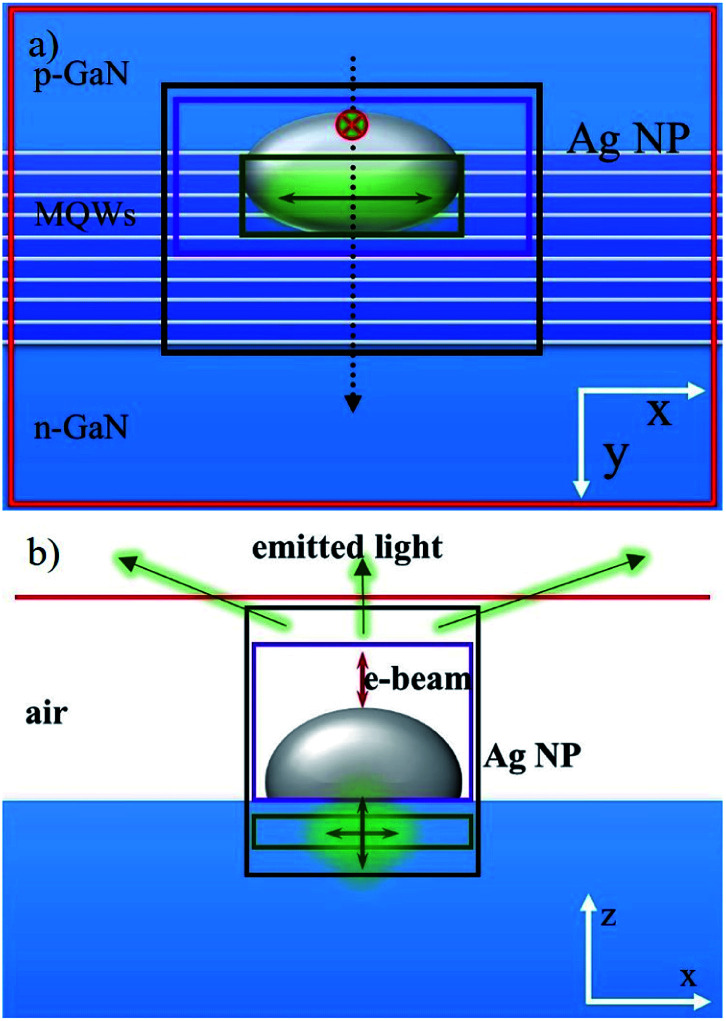
(a) The top view and (b) side view of the schematic structure in 3D FDTD simulation. The green, purple and black box monitors are used to collect the total radiated power by the q-dipole, the dissipated power and the scattered power by the Ag NP, respectively. The red plane monitor is used to record the radiated power from the top surface. The black dashed line corresponds to the e-beam scanning path.

To further understand the LSP enhanced CL intensity quantitatively and the LSP-QW coupling process related to the dipole orientation, 3D FDTD numerical method are used.^27^ In FDTD simulation, Maxwell's equations are solved in discretized space and time. According to the cross-sectional SEM image in Fig. 1(a), the simulation model is built as shown in Fig. 4. Each QW is represented by a series of point dipoles (q-dipoles) polarized within the QW-plane (*x*–*z* plane). Considering the symmetry of q-dipole orientation in the QW-plane, the polarized angle between the q-dipole orientation and *z*-axis is set to vary from 0° to 90°. Dipoles with the orientations of 0° and 90° are corresponding to radial and orbital dipoles in ref. 19. In addition, the space between the q-dipole and the surface is set to be 10 nm according to our previous work.^15^ The 10 nm spacer can achieve light emission enhancement in a wide range of the Ag NP size. In FDTD, the e-beam is modelled as a series of point dipoles with phase delay related to the e-beam velocity.^22^ Actually, the e-beam velocity only modifies the phase delay through a cosine function.^29^ This allows one to calculate all dipoles in one simulation or split these dipoles to smaller sub-simulations. Therefore, to simplify the model, the e-beam is represented by another point dipole (z-dipole) polarized along its trajectory (*z*-axis), as shown by a red dipole in Fig. 4. By moving the z-dipole successively along the black dashed line, which is corresponding to red line in Fig. 1(a), CL line scanning with the Ag NP can be simulated. Three power transmission boxes (green, purple and black) are used to record the radiated power by q-dipole, the dissipated power and the scattered power by the Ag NP, while a red plane monitor placed over the Ag NP is used to collect the power into the air. By default, the powers recorded by all monitors in one simulation are normalized to the sum of power from all sources (*P*_source_). For consistency, all calculated powers are renormalized by multiplying a correction factor of *P*_source_/*P*_0_, where *P*_0_ is the radiated power by q-dipole in a homogeneous environment (here GaN with a refractive index of 2.55). Specially, for the q-dipole, the radiated power enhancement (*P*_q-dipole_) is equal to the decay rate enhancement, or the Purcell factor (*F*_p_), which indicates the enhancement of spontaneous emission rate.^27,30–32^

According to the calculations in Fig. 3, without an Ag NP nearby, the QWs can be directly excited by the e-beam, where CL intensity is proportional to the intersecting volume with QW layers. As the e-beam impinges on the Ag NP, the LSP in the Ag NP is strongly excited by high-energy e-beam^21^ rather than by the electron–hole pair in QWs. Since the excitation by the e-beam is broadband optical sensitive and can induce both the LSP dipole and high order modes,^21–23^ the evanescent field is a superposed result of several modes and very strong. The dipoles with different polarization orientation and position coupled to LSP are simulated by Kuo *et al.*^20^ When the polarization orientation difference of the two dipoles varied from 10° to 180°, the radiated power enhancement changes smoothly. However, the misunderstanding information would appear when there is more constructive interference between the two dipoles without coupling to LSP. Therefore, the z-dipole induced LSP does not act as the imaging dipole to coupling with q-dipoles, but as a power supply through the evanescent field in this simulation. Moreover, to avoid interaction between the dipoles in the QWs with different orientations, only one q-dipole with a certain orientation is set up in a sub-simulation. Thus, the CL intensity can be obtained by the summation,

1 *I*_CL_ = ∑*η*_EQE_(*θ*,*r*_qw_)*P*_inj_(*r*_e_,*r*_qw_)

where *P*_inj_(*r*_e_,*r*_qw_) is the power injected to each QW position (*r*_qw_) at a certain e-beam impinging position (*r*_e_) and *η*_EQE_(*θ*,*r*_qw_), namely the external quantum efficiency (EQE) for each QW, is a function of the polarized angle (*θ*) and QW position (*r*_qw_). The EQE for each QW can be calculated by *η*_EQE_ = *η*_IQE_*η*_LEE_, where internal quantum efficiency (*η*_IQE_) and light extraction efficiency (*η*_LEE_) satisfy the following relationship,^11,20^2
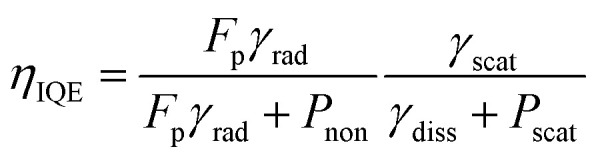
3
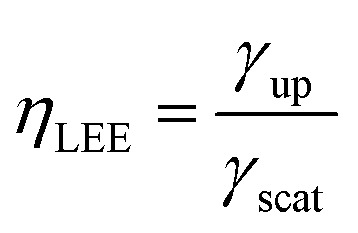
where the Purcell factor (*F*_p_), dissipation rate (*γ*_diss_), scattering rate (*γ*_scat_), light extraction upside rate (*γ*_up_) are recorded by the green, purple, black and red monitors (*P*_gBox_, *P*_pBox_, *P*_bBox_, *P*_up_) repectively. According to the temperature dependent PL measurement, the internal quantum efficiency is determined as 26%. Based on the IQE definition, the intrinsic ratio of non-radiative decay rate (*γ*_non_) to radiative decay rate (*γ*_rad_) is estimated to be 3 : 1.

**Fig. 5:**
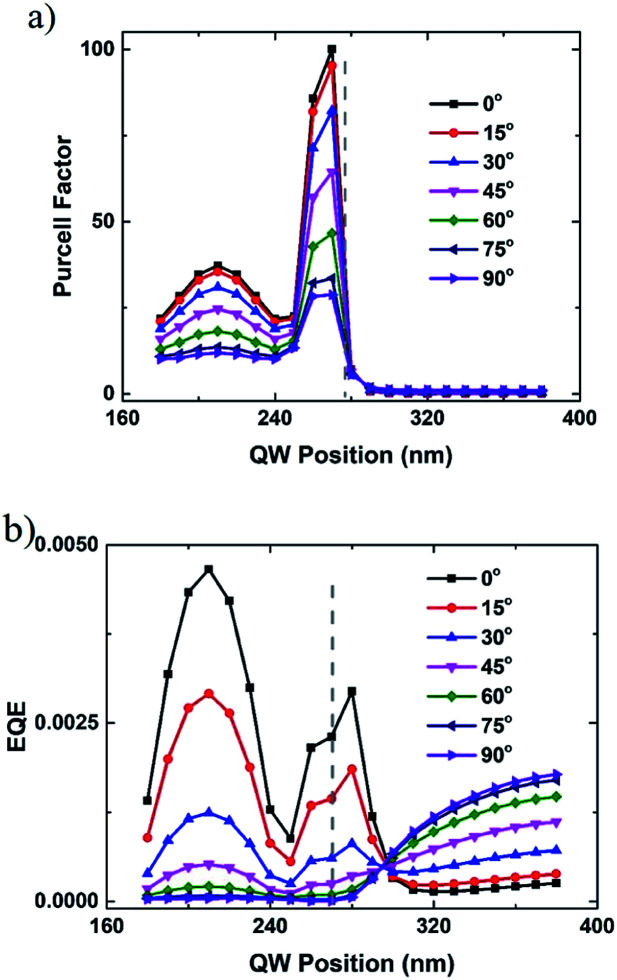
(a) Calculated Purcell factor (*F*_p_) and (b) the external quantum efficiency (EQE) for each QW with the different polarized angle.

Fig. 5 shows the calculated Purcell factor and EQE for each QW (q-dipole). The grey dashed line indicates the boundary of the Ag NP. The QWs under the Ag NP are on the left of the grey dashed line. In Fig. 5(a), the *F*_p_ for the q-dipole under the Ag NP is several tens of times larger than those away from the Ag NP. There are two *F*_p_ peaks at 210 and 270 nm, indicating that there is a strong coupling between the QWs and the LSP in the Ag NP region. As the polarized angle increases from 0 to 90°, the *F*_p_ for the q-dipole at 270 nm decreases monotonically from 100 to 28.7, while the *F*_p_ for the q-dipole at 210 nm decreases from 37.2 to 11.9. The radial dipole shows about 3.5 times higher coupling strength with LSP compared with the orbital one, which agrees with the report by Yang *et al.*^19^ Compared with the QW at 210 nm, the QW at 270 nm has the larger *F*_p_ due to the strong edge effect.^33^ In Fig. 5(b), the EQE for the QWs in the Ag NP region has two similar peaks, and decreases monotonically with the polarized angle increasing from 0 to 90°. However, the EQE for the QW at 210 nm is about 2 times larger than those for the QW at 270 nm. The dissipation rate is larger at 270 nm for the edge effect because of its larger absorption rate.^33^ In Fig. 5(b), the EQE for the QWs at above 300 nm increases significantly with the polarized angle increasing. It is found in Fig. 5(a) that the *F*_p_ reduces to 0.104 at 0°, while the *F*_p_ is about 1 at 90° for the QWs away from the Ag NP. On the other hand, Purcell factor is directly related to the local density of states (LDOS) which can be calculated from the imaginary part of the dyadic Green's function in the direction of the dipole at the dipole position.^27,31^ Compared with a dipole placed in a homogeneous dielectric (GaN) environment, the calculated imaginary part of the z-component of the electric field (Im(*E*_z_)) for the q-dipole with an 0°angle decreases 8.5 times at the q-dipole position. However, for the dipole with a 90° angle, the imaginary part of the corresponding x-component (Im(*E*_x_)) barely changes. Therefore, the 10-time decrease of the *F*_p_ for the q-dipole is attributed to the reflected electromagnetic wave by the GaN/air interface and its feedback effect on the q-dipole. From the results above, one can see that the QWs under the Ag NP with smaller polarized angle less than 30° have stronger LSP-QW coupling and larger EQE compared with the QWs away from the Ag NP (from 300 to 380 nm). The larger *F*_p_ and EQE for smaller polarized angle will give the higher weight to the light emission.

**Fig. 6:**
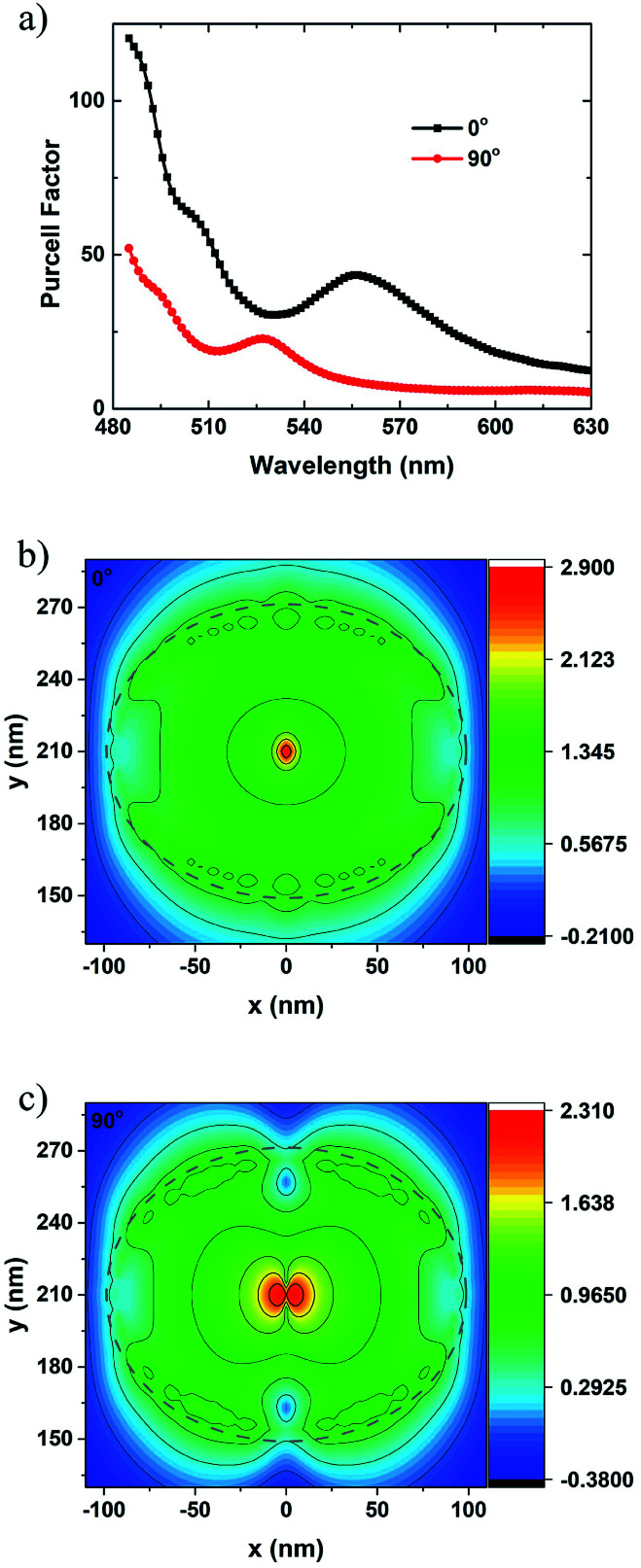
(a) Purcell factor curves for the q-dipoles at 210 nm with the polarized angles of 0° and 90°. 2D mappings of the electric field profile (on log scale) at the emission wavelength of 545 nm for the q-dipole with the angle of (b) 0° and (c) 90°. The color bars show the log scale of the electric field strength.

To get more details about the coupling process for dipoles with different polarization orientations, a plane monitor is placed under the bottom surface of Ag NP. The Purcell factor curves with respect to the wavelength are also calculated for these dipoles. Without loss of generality and to make it easier to analyze, Purcell factor for the dipoles at 210 nm (under the center of the Ag NP) with the polarized angles of 0° and 90° are plotted. As shown in Fig. 6(a), the resonant peak wavelengths are 555 and 525 nm for the LSP coupling with the dipoles with a 0° and 90° angle, respectively. The width of the resonant peak is about several tens of nanometres, as shown in Fig. 6(a). The 555 nm peak matches well with the emission wavelength of 545 nm, while the 525 nm peak is a bit far away from 545 nm. The coupling strength between q-dipole with a 0° angle and LSP is stronger than that with a 90° angle in the whole spectral range. Even so, the Purcell factor can reach about 10 at 545 nm for the q-dipole with a 90° angle. To classify the LSP modes, two-dimensional (2D) mappings of the electric field profile are calculated using the monitor as mentioned above. It is found that the 2D mapping patterns of the electric field are nearly unchanged within a wavelength range of ±30 nm away from the resonant peak wavelengths, as shown in Fig. 6(b) and (c). It is notable that the LSP mode excited by the q-dipole with a 0° angle shows a dipole mode characteristic dominated by the longer resonant peak at 555 nm as shown in Fig. 6(b).^19^ Similarly, a quadrupole mode characteristic dominated by the shorter resonant peak at 525 nm excited by the q-dipole with a 90° angle is observed, as shown in Fig. 6(c). As for the q-dipole with an arbitrary angle between 0° and 90°, the coupling modes can be regarded as the combination of these two basic coupling modes. It is notable that the electric field in Fig. 6(b) can be enhanced by a factor of 16 compared with that in Fig. 6(c), indicating again that the QWs with smaller polarized angle have stronger LSP-QW coupling. Since dipole mode coupling is stronger and can radiate light into air more easily,^12,19^ higher EQE with smaller polarized angle as discussed above can be proved. Recently, the LSP modes are also illustrated by far-field patterns.^34^ It is noticed that the LSP mode can be well interpreted with the combination of near-field and far-field patterns.

**Fig. 7:**
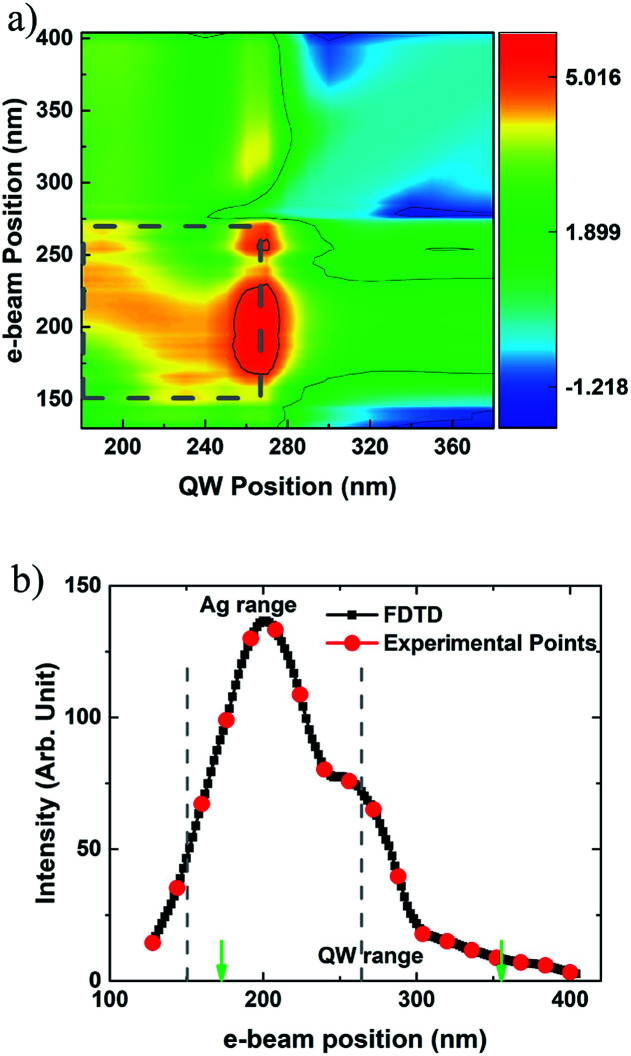
(a) Calculated power distribution (*P*_inj_) on log scale in the QW region injected by z-dipole induced LSP, and (b) calculated CL intensity for each e-beam position with a step of 2 nm (black) and corresponding experimental points (red). The color bar shows the log scale of the normalized electric field.

According to eqn (1), the injected power, *P*_inj_(*r*_e_,*r*_qw_) at each QW position for each e-beam excitation needs to be calculated. Fig. 7(a) shows power distribution in the QWs region which is excited by e-beam point by point along the red line in Fig. 1(a). The power is normalized by the injected power without the Ag NP. The grey dashed rectangle indicates the range of the Ag NP. As mentioned above, the LSP is induced by the e-beam and the evanescent field is mainly distributed in the Ag NP region. The strongest field is at 270 nm, which is the edge of Ag NP. In the Ag NP region, there is a secondary peak varying with the e-beam impinging position changing. The injected power at the edge of the Ag NP is about 1.5 orders of magnitude higher than that of the secondary peak. And the injected power is two orders of magnitude higher in the center area of the Ag NPs than those after the position of 300 nm. Moreover, when the e-beam moves to the positions far away from the Ag NP, the power injected to QWs located at the edge and center area is still higher. Therefore, the energy is transferred to the QWs through the LSP strongly induced by the e-beam, especially in the near-field vicinity of the Ag NP. As for the excitation away from the Ag NP, the injected power is divided into two parts: to excite the QWs directly and to excite the LSPs in the Ag NP.

After the EQE and *P*_inj_(*r*_e_,*r*_qw_) are calculated, CL line scanning result with the Ag NP can be calculated. The black line in Fig. 7(b) shows the simulated results and corresponding experimental points for CL line scanning with the Ag NP. The simulated line scanning step is 2 nm. Both CL enhancement and the shape of the curve show a good agreement. When the e-beam moves away from the Ag NP, the calculated intensity also decreases exponentially but much faster than the experimental values. Experimentally, QWs within a certain volume rather than QWs aligned in a line used in the simulation above will be excited and emit photons.^26^ However, due to the computation capacity and the approximations used in FDTD including dipole approximation for the QW and the e-beam,^2^ the location and polarization of dipole approximation and the simplification of LED structure and so on, the calculated curve appears to be more steep and drops faster. According to ref. 34, different e-beam impinging points show different far-field patterns. It will be concerned how the near field induced by e-beam affect the directionality of far field in the further work.

As discussed above, the simulation for CL line scanning can be calculated by dividing the calculation into two steps, namely calculating the power distribution injected by z-dipole induced LSP and the EQE for each q-dipole respectively. The LSP-QW coupling is strongly dependent on the polarized angle of the radiating dipoles. For the QW at the same position within the near-field vicinity of the Ag NP, the dipoles with smaller polarized angle leads to stronger lower-order LSP-QW coupling mode and larger output light intensity. It is predicted that if a smaller polarized angle (named as radial dipole) is obtained such as metallic NPs embedded in the samples etched through QWs or core–shell structured LEDs, several times enhancement of light emission could be achieved compared with that with the orbital dipole samples. To further enhance the emission, the etched through QWs should have small lateral size to satisfy for the near field conditions.

## Conclusions

In summary, we fabricated the LSP-QW coupled sample by placing an Ag NP on the cross-section of the QWs, where the QW-plane was perpendicular to the bottom surface of the Ag NP. The light emission enhancement by a factor of 6.1 was obtained using the ultra-high spatial resolution CL technique. Total energy loss profile for the GaN were simulated using CASINO to approximate electron–hole pair generation, which proved to be a good fit to the result of CL line scan without the Ag NP. To analyze the LSP-QW coupling process, a two-step simulation was carried out. The calculated result showed that dipoles with smaller polarized angle had larger Purcell factor and contributed more to the light emission enhancement through coupling with the lower-order LSP mode.

## Conflicts of interest

There are no conflicts to declare.

## Supplementary Material

## References

[cit1] O'Donnell K. P., Auf der Maur M., Di Carlo A., Lorenz K. (2012). Phys. Status Solidi RRL.

[cit2] Kuo Y., Ting S. Y., Liao C. H., Huang J. J., Chen C. Y., Hsieh C., Lu Y. C., Chen C. Y., Shen K. C., Lu C. F., Yeh D. M., Wang J. Y., Chuang W. H., Kiang Y. W., Yang C. C. (2011). Opt. Express.

[cit3] Lu C. H., Lan C. C., Lai Y. L., Li Y. L., Liu C. P. (2011). Adv. Funct. Mater..

[cit4] Jiang S., Hu Z., Chen Z. Z., Fu X. X., Jiang X. Z., Q Jiao Q., Yu T. J., Zhang G. Y. (2013). Opt. Express.

[cit5] Cho C. Y., Park S. J. (2016). Opt. Express.

[cit6] Okada N., Morishita N., Mori A., Tsukada T., Tateishi K., Okamoto K., Tadatomo K. (2017). J. Appl. Phys..

[cit7] Zhu S. C., Yu Z. G., Liu L., Yang C., Cao H. C., Xi X., Li J. M., Zhao L. X. (2017). Opt. Express.

[cit8] Okamoto K., Funato M., Kawakami Y., Tamada K. (2017). J. Photochem. Photobiol., C.

[cit9] Gontijo I., Boroditsky M., Yablonovitch E., Keller S., Mishra U. K., DenBaars S. P. (1999). Phys. Rev. B: Condens. Matter Mater. Phys..

[cit10] Jayabharathi J., Sujatha P., Thanikachalam V., Jeeva P., Panimozhi S. (2017). Ind. Eng. Chem. Res..

[cit11] Dionne J. A., Sweatlock L. A., Atwater H. A., Polman A. (2005). Phys. Rev. B: Condens. Matter Mater. Phys..

[cit12] Sun G., Khurgin J. B. (2010). Appl. Phys. Lett..

[cit13] Su C. Y., Lin C. H., Yao Y. F., Liu W. H., Su M. Y., Chiang H. C., Tsai M. C., Tu C. G., Chen H. T., Chiang Y. W., Yang C. C. (2017). Opt. Express.

[cit14] Kim J. M., Kim S. K., Hwang S. W., Kim C. O., Shin D. H., Kim J. H., Jang C. W., Kang S. S., Hwang E., Choi S. H., Gohary S. H. E., Byun K. M. (2018). Nanotechnology.

[cit15] Jiang S., Chen Z. Z., Fu X. X., Jiao Q. Q., Feng Y. L., Yang W., Ma J., Li J. Z., Jiang S. X., Yu T. J., Zhang G. Y. (2015). IEEE Photonics Technol. Lett..

[cit16] Fadil A., Iida D., Chen Y. T., Ou Y. Y., Kamiyama S., Ou H. Y. (2016). J. Lumin..

[cit17] Akselrod G. M., Argyropoulos C., Hoang T. B., Ciracì C., Fang C., Huang J., Smith D. R., Mikkelsen M. H. (2014). Nat. Photonics.

[cit18] Kuo Y., Chen H. T., Chang W. Y., Chen H. S., Yang C. C., Kiang Y. W. (2014). Opt. Express.

[cit19] Kuo Y., Chang W. Y., Chen H. S., Kiang Y. W., Yang C. C. (2013). Appl. Phys. Lett..

[cit20] Kuo Y., Lin C. H., Chen H. S., Hsieh C., Tu C. G., Shih P. Y., Chen C. H., Liao C. H., Su C. Y., Yao Y. F., Chen H. T., Kiang Y. W., Yang C. C. (2015). Jpn. J. Appl. Phys..

[cit21] Coenen T., Schoen D. T., Brenny B. J. M., Polman A., Brongersma M. L. (2016). Phys. Rev. B: Condens. Matter Mater. Phys..

[cit22] Maiti A., Maity A., Satpati B., Large N., Chini T. K. (2017). J. Phys. Chem. C.

[cit23] Lloyd J. A., Ng S. H., Liu A. C. Y., Zhu Y., Chao W., Coenen T., Etheridge J., Gómez D. E., Bach U. (2017). ACS Nano.

[cit24] Estrin Y., Rich D. H., Keller S., DenBaars S. P. (2015). J. Appl. Phys..

[cit25] Gao N., Huang K., Li J. C., Li S. P., Yang X., Kang J. Y. (2012). Sci. Rep..

[cit26] Toth M., Phillips M. R. (2010). Scanning.

[cit27] FDTD Solutions (v8.17), Lumerical, Inc., Canada, 2017

[cit28] de Abajo F. J. G. (2010). Rev. Mod. Phys..

[cit29] Cao Y., Manjavacas A., Large N., Nordlander P. (2015). ACS Photonics.

[cit30] Purcell E. M. (1946). Phys. Rev..

[cit31] Novotny L. and Hecht B., Principles of Nano-Optics, Cambridge University Press, 2006

[cit32] Faraon A., Barclay P. E., Santori C., Fu K. M. C., Beausoleil R. G. (2011). Nat. Photonics.

[cit33] Xu X. B., Luo J. S., Liu M., Wang Y. Y., Yi Z., Li X. B., Yi Y. G., Tang Y. J. (2015). Phys. Chem. Chem. Phys..

[cit34] Coenen T., Arango F. B., Koenderink A. F., Polman A. (2014). Nat. Commun..

